# Sarcoidosis in Melanoma Patients: Case Report and Literature Review

**DOI:** 10.3390/cancers7020821

**Published:** 2015-06-15

**Authors:** Bryce D. Beutler, Philip R. Cohen

**Affiliations:** 1 School of Allied Health Sciences, University of Nevada, Las Vegas, 1060 Wiegand Road, Encinitas, CA 92024, USA; 2 Department of Dermatology, University of California San Diego, 10991 Twinleaf Court, San Diego, CA 92131, USA

**Keywords:** cancer, cutaneous, malignancy, melanoma, sarcoid, sarcoidosis, solid tumors

## Abstract

Sarcoidosis is a systemic inflammatory disease characterized by the development of noncaseating granulomas in multiple organ systems. Many hematologic malignancies and solid tumors, including melanoma, have been associated with sarcoidosis. We describe the clinical and pathologic findings of a 54-year-old man with melanoma-associated sarcoidosis. In addition, we not only review the literature describing characteristics of other melanoma patients with sarcoidosis, but also the features of melanoma patients with antineoplastic therapy-associated sarcoidosis. Sarcoidosis has been described in 80 melanoma patients; sufficient information for analysis was provided in 39 of these individuals. In 43.6% of individuals (17 out of 39), sarcoidosis was directly associated with melanoma; in 56.4% of oncologic patients (22 out of 39), sarcoidosis was induced by antineoplastic therapy that had been administered for the treatment of their metastatic melanoma. The discovery of melanoma preceded the development of sarcoidosis in 12 of the 17 (70.5%) individuals who did not receive systemic treatment. Pulmonary and/or cutaneous manifestations of sarcoidosis were common among both groups of patients. Most patients did not require treatment for sarcoidosis. Melanoma patients—either following antineoplastic therapy or without systemic treatment—may be at an increased risk to develop sarcoidosis. In antineoplastic therapy naive melanoma patients, a common etiologic factor—such as exposure to ultraviolet light—may play a role in their developing melanoma and sarcoidosis.

## 1. Introduction

Sarcoidosis is a systemic inflammatory disease in which noncaseating granulomas develop in multiple organ systems. The lungs and intrathoracic lymph nodes are most commonly affected. However, manifestations of sarcoidosis can also involve other organs as well as the central nervous system. 

Hematologic malignancies and solid tumors, including melanoma, have been associated with sarcoidosis. Indeed, oncologic patients have an increased risk of developing sarcoidosis and sarcoidosis patients have an increased risk of developing a malignancy. In some individuals, sarcoidosis and cancer have been diagnosed concurrently or within 12 months of one another. In other patients, however, systemic antineoplastic therapy may have contributed to the subsequent development of sarcoidosis. 

We describe a man who presented with two skin lesions; they were biopsied and showed melanoma *in situ* and cutaneous sarcoidosis. We also review the literature describing characteristics of other melanoma patients with sarcoidosis. 

## 2. Results 

In March 2014, a 54-year-old man with a history of cardiomyopathy presented for evaluation with a chief complaint of intermittent tinnitus, dizziness, and nausea of one month duration. He reported that the symptoms improved with meclizine and denied any associated otalgia. In addition, he complained of intermittent numbness and shooting pain in his left leg. However, his neurological examination was normal. The patient agreed to return for nerve conduction studies and an electromyogram at a later date.

**Figure 1:**
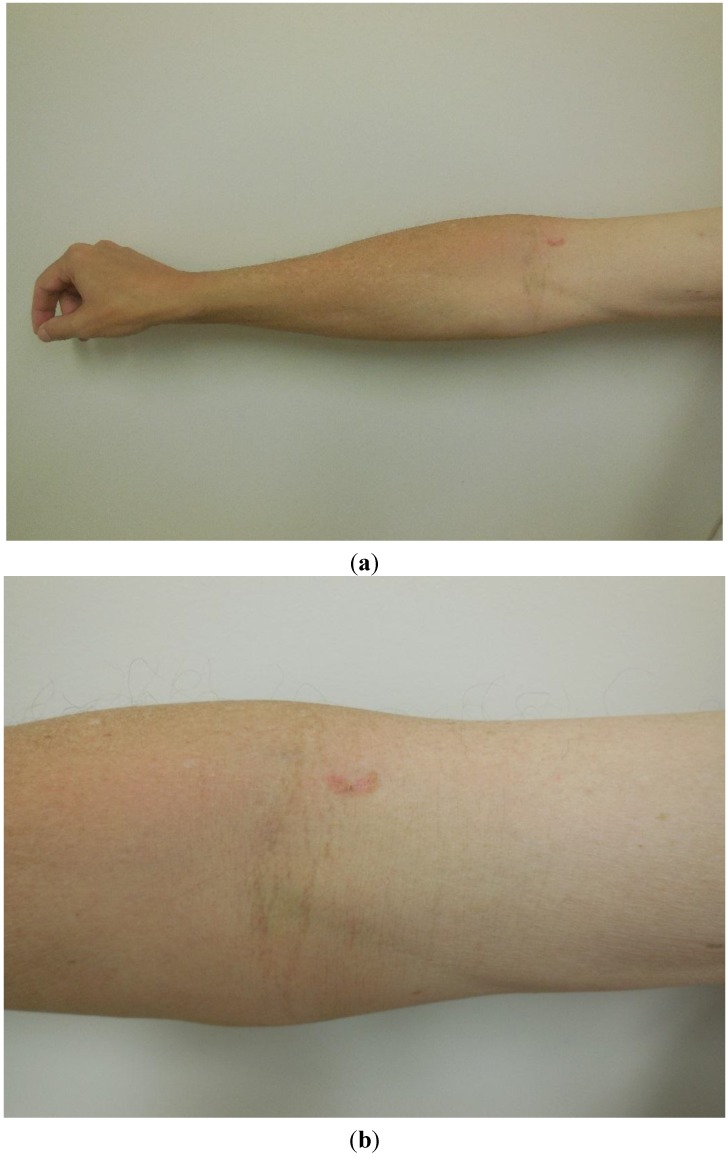
Distant (**a**) and close (**b**) views of a red-brown colored plaque involving the right antecubital fossa of a 54-year-old man. The lesion was later diagnosed as melanoma *in situ*.

**Figure 2:**
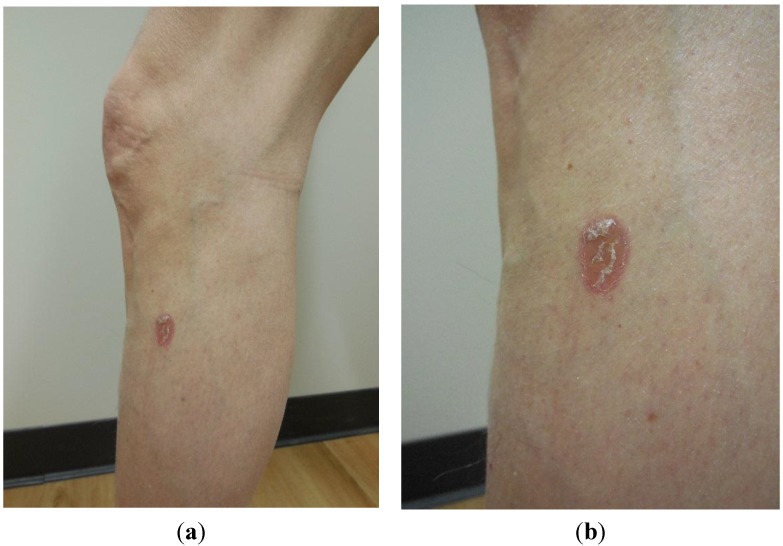
Distant (**a**) and close (**b**) views of a scaly annular violaceous plaque on the left calf of a 54-year-old man. The lesion was later diagnosed as cutaneous sarcoidosis.

In May 2014, he sought evaluation for lesions on his face, chest, and extremities. A complete head-to-toe skin examination not only revealed multiple actinic keratoses at these locations, but also a 1.5 × 0.5 centimeter red-brown colored plaque involving the right antecubital fossa that had been present for at least one year (Figure 1). In addition, there was a 2 × 1 centimeter scaly annular violaceous plaque on his left calf that had appeared approximately two months earlier (Figure 2); he had a similar appearing lesion in the same location a year earlier that was treated with a topical corticosteroid cream and resolved. The actinic keratoses were treated with liquid nitrogen cryotherapy. The plaques on his right antecubital fossa and left calf were biopsied.

**Figure 3:**
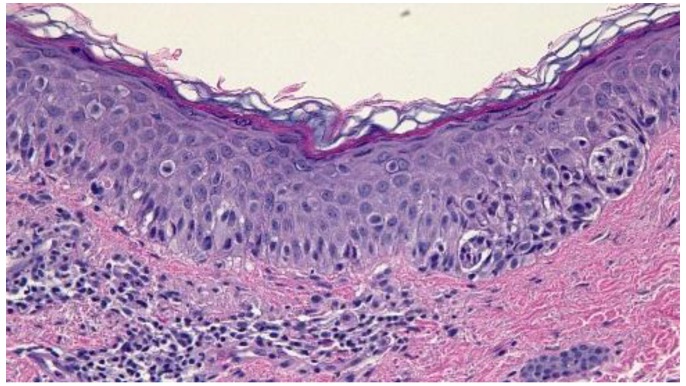
High magnification view of a sample of a lesion taken from the right antecubital fossa of a 54-year-old man. A broad proliferation of moderately atypical melanocytes arranged as both irregular-sized nests and single cells along the dermal-epidermal junction can be observed. [Hematoxylin and eosin; ×40].

**Figure 4:**
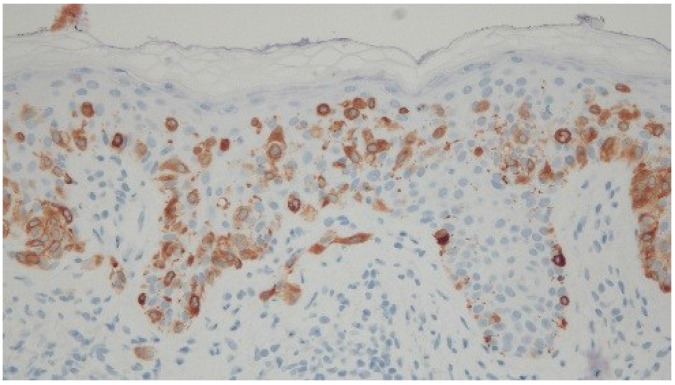
High magnification view of a sample of a lesion taken from the right antecubital fossa of a 54-year-old man. Areas of contiguous single cell growth as well as a high level pagetoid spread are highlighted by Melanoma Antigen Recognized by T Cell 1 (MART-1) immunohistochemical staining. The pagetoid melanocytes stained negatively for cytokeratin Anion Exchanger Isoforms 1-3 (AE1/3). A small focus of bland-appearing nevoid melanocytes can also be seen in the superficial papillary dermis. [Melanoma Antigen Recognized by T Cell 1; ×40].

Histologic examination of the tissue from his right antecubital fossa revealed a broad proliferation of moderately atypical melanocytes arranged as both irregular-sized nests and single cells along the dermal-epidermal junction (Figure 3). Areas of contiguous single cell growth as well as a high level pagetoid spread were highlighted by Melanoma Antigen Recognized by T Cell 1 (MART-1) immunohistochemical staining. The pagetoid melanocytes stained negatively for cytokeratin Anion Exchanger Isoforms 1-3 (AE1/3). A small focus of bland-appearing nevoid melanocytes could also be seen in the superficial papillary dermis (Figure 4). The findings were consistent with melanoma *in situ* arising in the context of a compound nevus. The lesion was excised without complication or recurrence.

**Figure 5:**
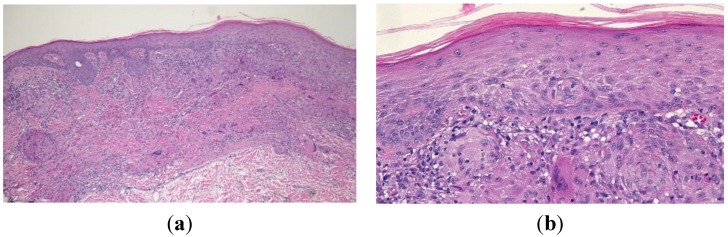
Low (**a**) and high (**b**) magnification views of the sarcoidal granuloma taken from the left calf of a 54-year-old man. Low magnification view (a) shows hyperplasia and hyperkeratosis in the overlying epidermis as well as a lichenoid lymphocytic infiltrate at the dermal-epidermal junction. Langerhans cells and spindle-shaped interstitial histiocytes can be observed in the high magnification view (b). [Hematoxylin and eosin; a = ×10, b = ×40].

Histologic examination of the skin from his left calf revealed sarcoidal granulomas comprised of epithelioid-appearing histiocytes within the superficial dermis. Langerhans cells were present. The overlying epidermis demonstrated areas of hyperplasia and hyperkeratosis. A lichenoid lymphocytic infiltrate was also observed at the dermal-epidermal junction. Spindle-shaped interstitial histiocytes could be seen in the upper portions of the dermis. The spindle-shaped cells as well as the epithelioid component stained positively for CD68 and negatively for cytokeratin AE1/3 (Figure 5). Correlation of the clinical presentation of the lesion and the pathology was consistent with cutaneous sarcoidosis. The patient applied betamethasone dipropionate 0.05% cream twice a day for two weeks and the lesion resolved.

In July 2014, the patient returned for follow-up. Prompted by the discovery of sarcoidosis in the skin, a systemic evaluation was subsequently performed. Laboratory results for the following studies were negative or normal: angiotensin-coverting enzyme (22 mcg/L), erythrocyte sedimentation rate (7 mm/h), serum sodium (139 mEq/L), serum potassium (3.9 mEq/L), serum calcium (9.5 mg/dL), aspartate aminotransferase (16 IU/L), alanine aminotransferase (13 IU/L), and serum glucose (114 mg/dL). 

Pulmonary evaluation included a chest roentgenogram and a chest computed tomography scan. The former was normal. However, the scan revealed multiple scattered subpleural and pleural nodularities. Mild ground-glass opacity surrounding a bronchovascular bundle in the left upper lobe was also observed. In addition, there was reticulation and micronodularity in the left lower lobe. These findings were diagnostic of stage 1 pulmonary sarcoidosis. 

His neurologic symptoms of intermittent tinnitus, dizziness, nausea, and left leg numbness had not improved. Based on the diagnosis of systemic sarcoidosis (with pulmonary and cutaneous involvement), he was evaluated for neurosarcoidosis. Nerve conduction studies and an electromyogram were performed; both were normal. A lumbar puncture revealed elevated protein (65 mg/dL) and glucose (77 mg/dL) in the cerebral spinal fluid; however, no inflammatory cells were present. An orbit computed tomography scan showed a small amount of mucosal thickening along the left auditory canal extending over part of the tympanic membrane and a head computed tomography scan revealed inflammation of the external auditory canal and tympanic membrane. The central nervous system evaluation was not conclusive, but the computed tomography scans of the orbit and head were suggestive of a possible diagnosis of neurosarcoidosis.

Treatment was initiated with 40 milligrams of prednisone daily; his neurological symptoms progressively improved. The concurrent discovery of skin cancer and his systemic condition was also diagnosed as melanoma-associated sarcoidosis. 

## 3. Discussion

### 3.1. Cutaneous Manifestations of Sarcoidosis

Patients with sarcoidosis can have disease-associated skin lesions. Skin involvement in sarcoidosis is classified as either “nonspecific” or “specific”. Nonspecific skin lesions are often associated with acute sarcoidosis and do not demonstrate sarcoidal granulomas. In contrast, disease-specific skin lesions are usually associated with chronic sarcoidosis and reveal noncaseating granulomas in the dermis, subcutaneous fat, or both [1]. 

Erythema nodosum is the most common nonspecific sarcoidosis-associated skin lesion [2]. Patients with erythema nodosum typically present with tender, erythematous nodules of one to five centimeters in diameter on the anterior surfaces of the lower legs. Fever, fatigue, and arthralgia may also be present. The condition is usually self-limiting and resolves within two to six weeks without treatment. However, non-steroidal anti-inflammatory drugs and antihistamines may be used for symptomatic relief [3]. Other nonspecific lesions associated with sarcoidosis include erythema multiforme, nummular eczema, and calcinosis cutis [4].

Specific lesions associated with sarcoidosis are pleomorphic in appearance. They may present as papules, plaques, subcutaneous nodules, or hypopigmented patches. Indeed, cutaneous sarcoidosis is sometimes referred to as “the great imitator” because it can exhibit so many different lesion morphologies [5]. 

Papules are the most common type of sarcoidosis-specific skin lesion [6]. Interestingly, papular sarcoidosis-associated eruptions are associated with a better prognosis when compared with other types of disease-specific lesions. The differential diagnosis for papular sarcoidosis lesions includes acne, angiofibromas, granuloma annulare, and lichen planus [1]. 

Lupus pernio typically presents as indurated purple facial plaques. However, the fingers may also be affected. It is most commonly seen in African American women with persistent sarcoidosis. Lupus pernio may not only mimic lupus erythematosus, but also rhinophyma if localized to the nose [1]. 

Subcutaneous nodules are a rare manifestation of sarcoidosis. However, individuals with malignancy-associated sarcoidosis who present with the new onset of subcutaneous nodules should be evaluated promptly in order to exclude metastasis. Subcutaneous nodules may also mimic epidermoid cysts and lipomas [1]. 

Ichthyosiform sarcoidosis is another rare cutaneous manifestation of sarcoidosis that appears identical to acquired ichthyosis—an asymptomatic scaly eruption that most commonly affects the lower extremities. Lesional biopsy not only shows pathologic changes of ichthyosis vulgaris, but also sarcoidal granulomas in the affected skin. Patients presenting with ichthyosiform sarcoidosis often develop systemic disease [7]. 

Specific lesions may also develop within scars. One 49-year-old woman who had previously had a melanoma excision presented with three firm subcutaneous nodules at the border of the melanoma scar on her left arm [8]. Pathologic examination showed naked noncaseating granulomas in the subcutaneous fat. In addition, laboratory testing revealed slightly elevated angiotensin-coverting enzyme, a feature highly consistent with sarcoidosis [9]. However, the patient declined treatment.

### 3.2. Neurosarcoidosis

Sarcoidosis affecting the central nervous system—neurosarcoidosis—is rare. However, neurological symptoms were observed in an oncologic patient with treatment-associated sarcoidosis [10]. Similar to our patient, his symptoms resolved following treatment with oral corticosteroids.

The clinical presentation of neurosarcoidosis is highly variable. For instance, our patient complained of intermittent tinnitus, dizziness, and nausea. In contrast, the 36-year-old man with melanoma and ipilimumab-induced neurosarcoidosis experienced persistent headaches [10]. Therefore, magnetic resonance imaging is an important diagnostic tool for assessing nervous system involvement in sarcoidosis. A magnetic resonance imaging scan of Murphy *et al.*’s patient showed abnormal enhancing tissue in the sella turcica and adjacent to the pituitary infundibulum. Other common magnetic resonance imaging findings in patients with neurosarcoidosis include leptomeningeal enhancement, intraparenchymal mass lesions, and/or hydrocephalus [11]. Unfortunately, our patient was unable to proceed with magnetic resonance imaging because he had a pacemaker; however, the computed tomography scan of the orbits and the head showed features that were suggestive of sarcoidosis.

### 3.3. Malignancy-Associated Sarcoidosis

The coexistence of sarcoidosis and malignancy was first observed nearly a century ago. In a 1917 report, Herxheimer described sarcoid reactions in patients with carcinomas of the breast, rectum, and cystic duct [12]. Pautrier subsequently observed the reverse temporal association: the development of Hodgkin’s disease in a patient with previously diagnosed sarcoidosis [13]. However, large-scale studies investigating a possible link between sarcoidosis and cancer would not be conducted until the 1970s.

In 1972, Brincker reviewed 1500 patients with hematologic malignancies and identified five individuals who subsequently developed systemic sarcoidosis [14]. Interestingly, the incidence of sarcoidosis in the general population is much lower: approximately five in 100,000 [15]. Although Brincker’s study was not designed to calculate the frequency of malignancy-associated sarcoidosis, his findings served as one of the earliest indications of a possible etiologic link between the two conditions. 

Two years later, Brincker and Wilbek investigated the incidence of malignancies in patients who had previously been diagnosed with sarcoidosis. In an analysis of 2544 patients with respiratory sarcoidosis, they found that lymphoma and lung cancer occurred significantly more frequently than would be expected in the general population [16]. A subsequent linkage analysis study by Reich *et al.* validated Brincker and Wilbek’s results and provided strong evidence for an association between sarcoidosis and cancer. Indeed, investigators concluded that “sarcoidosis and malignancy may be etiologically related in at least a quarter of cases in which both are present [17]”.

**Table 1:** Characteristics of patients with melanoma-associated sarcoidosis (MAS).

C	Dx: MM	Dx: SC	PM	CM	G	MM site	MM thickness	SC tx	R
1	22	30	+	+	F	Right leg	0.5 mm	HCQ	9
2	35	35	+	−	M	Left leg	4.0 mm	N	18
3	35	36	+	−	F	Left arm	0.37 mm	N	19
4	35	37	+	+	F	Thorax	0.76 mm	NR	20
5	36	44	+	−	F	Left arm	0.91 mm	NR	20
6	40	40	+	+	M	Left foot	5.5 mm	N	21
7	41	37	+	−	M	Left ear	4.0 mm	CCS	22
8	43	43	+	−	F	Left leg	1.2 mm	CCS	23
9	49	49	−	+	F	Left arm	NR	N	8
10	50	51	+	−	F	Right leg	3.0 mm	N	22
11	53	30	+	−	M	Right shoulder	1.1 mm	N	9
12	54	54	+	−	M	Abdomen	2.8 mm	N	9
13	54	54	+	+	M	Right arm	0.0 mm	CCS	CR
14	56	52	+	+	F	Right foot	0.4 mm	HCQ & CCS	9
15	63	63	+	−	F	Right ear	1.84 mm	N	24
16	68	65	+	−	F	Right heel	2.6 mm	N	25
17	81	76	+	−	F	Jaw	1.5 mm	CCS	9

Abbreviations: C = case; CCS = corticosteroids; CM = cutaneous manifestations of sarcoidosis; CR = current report; Dx: MM = age at melanoma diagnosis (years); Dx: SC = age at sarcoidosis diagnosis (years); F = female; G = gender; HCQ = hydroxychloroquine; M = male; MM site = primary site of melanoma lesion; MM thickness = Breslow thickness of melanoma lesion (millimeters); N = no treatment; NR = not reported; PM = pulmonary manifestations of sarcoidosis; R = reference number; SC tx = sarcoidosis treatment.

### 3.4. Sarcoidosis in Melanoma Patients

There are two clinical settings in which sarcoidosis may occur in melanoma patients. Individuals with melanoma who did not receive systemic antineoplastic therapy are described in Table 1 [8,9,18,19,20,21,22,23,24,25]; we will refer to these patients as having melanoma-associated sarcoidosis (MAS). In contrast, individuals with melanoma who did receive systemic antineoplastic therapy and subsequently developed sarcoidosis are described in Table 2 [9,10,20,23,26,27,28,29,30,31,32,33,34,35,36,37,38,39]; we will refer to these patients as having drug-associated sarcoidosis (DAS). In addition to the patients listed in Table 1 and Table 2, there are 41 reports of melanoma patients with sarcoidosis in which specific patient characteristics were not provided [40,41,42,43,44,45]. Therefore, sarcoidosis has been described in a total of 80 melanoma patients.

**Table 2:** Characteristics of patients with drug-associated sarcoidosis (DAS).

C	Dx: MM	Dx: SC	PM	CM	G	MM site	MM thickness	ANP therapy	SC tx	R
1	16	21	+	−	M	Eyebrow	NR	IFN-α	N	9
2	21	24	+	−	F	Axilla	2.7 mm	Vindesine	N	23
3	32	32	+	−	F	Left leg	1.82 mm	IFN-α	WD	26
4	35	49	+	−	M	Right calf	2.0 mm	Ipilimumab	N	27
5	36	37	+	−	M	Leg	NR	Ipilimumab	CCS	10
6	36	38	−	+	F	Left leg	NR	IFN α-2b	WD	28
7	36	38	+	−	M	Back	NR	Vemurafenib	N	29
8	42	44	−	+	M	Unknown primary	NR	Interferon α-2b	N	30
9	45	58	+	−	M	Right thumb	NR	IFN-β	WD	31
10	47	47	+	−	M	Thorax	2.95 mm	Peginterferon α-2a	WD	32
11	47	47	+	−	M	Unknown primary	NR	Peginterferon α-2b	WD	32
12	48	56	+	−	F	Left knee	NR	Ipilimumab	CCS	33
13	50	51	+	+	M	Left calf	5.04 mm	Interferon α-2b, peginterferon α-2a	WD	32
14	50	54	+	+	F	Scalp	1.45 mm	IFN-α	N	34
15	51	55	+	−	F	Left ankle	2.5 mm	Peginterferon α-2b	N	35
16	52	53	+	+	M	Left leg	3.2 mm	IFN-α	N	36
17	60	60	+	+	M	Left shoulder	4.0 mm	IFN-α	N	37
18	62	67	+	+	F	Back	1.5 mm	Ipilimumab	WD	9
19	65	66	+	−	F	Left leg	4.9 mm	IFN-α, dacarbazine	NR	20
20	67	72	+	+	F	Back	1.5 mm	Ipilimumab	N	38
21	68	68	−	+	M	Unknown primary	NR	Vemurafenib	HCQ & CCS	29
22	70	71	+	+	F	Right toe	NR	IFN α-2b	N	39

Abbreviations: ANP therapy = antineoplastic therapy; C = case; CCS = corticosteroids; CM = cutaneous manifestations of sarcoidosis; Dx: MM = age at melanoma diagnosis (years); Dx: SC = age at sarcoidosis diagnosis (years); F = female; G = gender; HCQ = hydroxychloroquine; IFN-α = interferon-alpha; IFN-β = interferon-beta; M = male; MM site = primary site of melanoma lesion; MM thickness = Breslow thickness of melanoma lesion (millimeters); N = no treatment; NR = not reported; PM = pulmonary manifestations of sarcoidosis; R = reference number; SC tx = sarcoidosis treatment; WD = drug withdrawn.

### 3.5. Melanoma-Associated Sarcoidosis (MAS)

There were 17 reports of MAS in which specific patient characteristics were described. A slight female predominance (65%) was observed. The age at melanoma diagnosis ranged from 22 to 81 years, with a median age of 49 years. Sarcoidosis and melanoma were diagnosed at least a year apart in ten out of 17 (58.8%) patients with MAS. However, both conditions were diagnosed simultaneously or within one year of one another in seven individuals. The interval between the diagnosis of melanoma and sarcoidosis ranged from 0 to 23 years, with a mean interval of approximately 3.5 years. Interestingly, 12 out of 17 (70.5%) patients with MAS presented first with melanoma and were later diagnosed with sarcoidosis. However, it is not uncommon for the onset of sarcoidosis to precede the development of melanoma. Indeed, some evidence suggests that there is an increased risk of melanoma among patients who were previously diagnosed with sarcoidosis [40].

Melanoma lesions most commonly developed on the extremities, but also appeared on the face, abdomen, thorax, and ear. The Breslow thickness was reported in 16 out of 17 patients and ranged from 0.0 millimeters to 5.5 millimeters; the mean Breslow thickness was approximately 1.91 millimeters. All 17 patients had surgical excision of their primary melanoma.

Pulmonary manifestations of sarcoidosis were present in 16 out of 17 patients. Five patients presented with both pulmonary sarcoidosis and sarcoidal skin lesions. In addition, one patient developed cutaneous sarcoidosis with no pulmonary involvement.

In most patients, the diagnosis of pulmonary sarcoidosis was established based on chest roentgenography and/or computed tomography. Bilateral hilar lymphadenopathy was a common finding. Pulmonary infiltration and fibrosis were also present among patients who had severe disease. Several patients underwent a mediastinal lymph node biopsy in order to definitively exclude metastatic disease. Interestingly, our patient had a normal chest roentgenogram; a chest computed tomography scan was required to confirm the diagnosis of systemic sarcoidosis. Therefore, a combination of multiple diagnostic tests—including roentgenography, computed tomography, and biopsy—may sometimes be required for the assessment of suspected pulmonary sarcoidosis. 

All six MAS patients with cutaneous manifestations of sarcoidosis presented with specific skin lesions, including subcutaneous nodules and violaceous plaques. Sarcoidosis resolved spontaneously in the majority of individuals diagnosed with MAS. However, approximately 40% of patients required pharmacological therapy for symptomatic relief—either corticosteroids (four patients), hydroxychloroquine (one patient), or both (one patient).

### 3.6. Drug-Associated Sarcoidosis (DAS)

There were 22 reports of DAS in which specific patient characteristics were described. In contrast to patients with MAS, the female-to-male ratio was approximately equal (45% female and 55% male). The age at melanoma diagnosis ranged from 16 to 70 years, with a median age of 47.5 years. Sarcoidosis and melanoma were diagnosed at least a year apart in 17 out of 22 (77.2%) patients with DAS. However, both conditions were diagnosed simultaneously or within one year of one another in five individuals. The interval between the diagnosis of melanoma and sarcoidosis ranged from 0 to 14 years, with a mean interval of approximately 3.3 years. 

Melanoma lesions appeared on the extremities, face, back, digits, and scalp. The primary site of melanoma was unknown in two individuals with widespread metastases. The Breslow thickness was reported in 12 out of 22 patients and ranged from 1.45 millimeters to 5.04 millimeters; at approximately 2.80 millimeters, the mean Breslow thickness was substantially greater in patients with DAS than in those with MAS.

Pulmonary manifestations of sarcoidosis occurred in 19 of the 22 patients with DAS. Chest roentgenography, computed tomography, and/or positron emission tomography were used in conjunction with mediastinal lymph node biopsy in order to exclude melanoma metastasis and establish a diagnosis of pulmonary sarcoidosis. Bronchoscopy with bronchoalveolar lavage was also performed in several patients. In addition, an endobronchial biopsy was required to identify noncaseating granulomas in patients with suspected upper respiratory tract involvement. 

Specific sarcoidal skin lesions were present in ten patients with DAS. Seven patients had both pulmonary and cutaneous sarcoidosis; three patients presented with cutaneous manifestations only. The lesions were pleomorphic in appearance and included maculopapular eruptions and subcutaneous nodules. Notably, none of the patients developed erythema nodosum or any other nonspecific sarcoidal skin lesion.

Over 60% of the patients who developed DAS were being treated with interferons. Other anti-cancer drugs that were linked to sarcoidosis included ipilimumab, vemurafenib, and vindesine. Most patients were able to continue receiving antineoplastic therapy after the onset of sarcoidosis. However, treatment was discontinued in seven patients who developed severe symptoms. In addition, three individuals required treatment for sarcoidosis—either corticosteroids (two patients) or combination therapy with corticosteroids and hydroxychloroquine (one patient). 

### 3.7. Pathogenesis of Cancer in Sarcoidosis Patients

#### 3.7.1. Autoimmune Mechanism 

An autoimmune mechanism involving T cells—specifically TH1 cells—and tumor necrosis factor alpha plays a fundamental role in the development of sarcoidosis [37]. However, the mechanism of pathogenesis for sarcoidosis in melanoma patients is unknown. 

#### 3.7.2. Ultraviolet Radiation Mechanism

Both melanoma and nonmelanoma skin cancers are associated with sarcoidosis. Therefore, ultraviolet radiation could conceivably represent a common etiologic factor [40]. Interestingly, the relationship between tumor necrosis factor alpha and ultraviolet light lends credence to the idea that sun exposure plays a central role in the development of melanoma-associated sarcoidosis. Researchers found that ultraviolet light not only induces cellular damage, but also upregulates tumor necrosis factor alpha, which in turn promotes granuloma formation [22,46]. However, further investigation is necessary in order to definitively understand the nature of the relationship between melanoma and sarcoidosis.

#### 3.7.3. Immunologic Mechanism

Malignancy-associated sarcoidosis has been postulated to have an immunologic origin [20]. A study of over 10,000 hospitalized sarcoidosis patients showed a 40% excess in the incidence of cancer occurring within the first year of hospitalization [47]. Notably, in this cohort, the risk of cutaneous squamous cell carcinoma was markedly increased. 

Patients with previously diagnosed sarcoidosis have an increased risk of cancer. In addition, oncologic patients with sarcoidosis have a worse prognosis [48]. Therefore, immune dysregulation may play a role in the development of both sarcoidosis and cancer. It has been postulated that the onset of sarcoidosis could conceivably be attributed to a tumor-antigen-induced cell-mediated response [20].

A decreased incidence of lung cancer was observed among men who had been hospitalized for sarcoidosis [49]. The investigators hypothesized that the men who had been diagnosed with sarcoidosis subsequently quit smoking, thus reducing their risk of developing lung cancer. Their hypothesis was supported by the observation that there was also a decrease in the incidence of other tobacco-related cancers among this cohort. 

In addition, researchers in this study also noted that the incidence of many malignancies—including cancers of the colon, kidney, and rectum—was significantly increased among patients who had been hospitalized for sarcoidosis [49]. Other studies have also observed similar findings [16,47,48,50]. Hence, investigators proposed that immune dysregulation or an unknown etiologic factor or both were possible mechanisms of pathogenesis for malignancy-associated sarcoidosis [49].

#### 3.7.4. Inflammation and Antigen Response Mechanism

Chronic inflammation and impaired antigen response have been suggested to promote the occurrence of cancer in sarcoidosis patients [51]. Myeloid dendritic cells, which play a central role in tumor immune surveillance, are thought to be involved in this response. However, further research is required to elucidate the role of immune dysregulation in malignancy-associated sarcoidosis.

#### 3.7.5. Antineoplastic Therapy Mechanism 

Antineoplastic therapies—including interferons, cisplatin, and interleukin 2—have previously been established to be associated with sarcoidosis [50]. Modulation of macrophage and T cell activity is the putative cause of interferon-induced sarcoidosis; specifically, interferon-alpha promotes granuloma formation by stimulating TH1 lymphocytes [37]. Similarly, ipilimumab promotes T cell activation and may reduce self-tolerance to normal tissues in some patients [33]. 

The other two antineoplastic therapies that have been linked to DAS—vemurafenib and vindesine—do not affect melanoma immunologically. Vemurafenib is a B-Raf enzyme inhibitor that interrupts the mitogen-activated protein kinase/extracellular-signal-regulated kinase (MAPK/ERK) pathway. Indeed, vemurafenib-related dysregulation of this pathway contributes to the development of sarcoidosis [29]. Vindesine is an anti-mitotic agent; currently, a plausible mechanism by which vindesine might induce sarcoidosis remains to be determined. Therefore, it is possible that the association between vindesine and sarcoidosis is coincidental.

### 3.8. Diagnostic Pitfalls: Mimickers of Sarcoidosis 

#### 3.8.1. Sarcoid-Like Reactions

In addition to sarcoidosis, oncologic patients may also develop sarcoid-like reactions. They are also sometimes referred to as either sarcoidal reactions or sarcoid reactions. In contrast to sarcoidosis, which is a systemic condition with or without associated skin lesions, patients with sarcoid-like reactions demonstrate sarcoidal granulomas—most commonly in the lungs, skin, and/or spleen—but do not fulfill the criteria for systemic sarcoidosis. 

Subtypes of sarcoid-like reactions have been described. The subtypes can be distinguished by the causative agent associated with the reaction. Infectious pathogens associated with sarcoid-like reaction include hepatitis viruses, mycobacteria, and propionibacteria. Non-infectious agents that may induce sarcoid-like reaction include tumor antigens and inorganic substances [5,52].

Similar to sarcoidosis, sarcoid-like reactions have also been associated with malignancies. These cancers include not only hematologic malignancies, but also solid tumors [50]. In patients with malignancy-associated sarcoid-like reaction, sarcoidal granulomas can also develop within the locoregional draining lymph nodes or the stroma surrounding the neoplasm [20]. However, in a 48-year-old Japanese woman with malignant melanoma, the neoplasm originated on her left thigh while the sarcoid-like reaction manifested both at adjacent and distant body sites, including subcutaneous nodules on her left arm [53].

#### 3.8.2. Infectious Diseases Mimicking Sarcoid-Like Reaction

In addition to neoplastic disease, sarcoid-like reactions have also been noted in patients with infections. A 35-year-old man who presented with facial sarcoidal granulomatous plaques was suspected to have systemic sarcoidosis. Subsequent imaging studies were consistent with a diagnosis of pulmonary sarcoidosis, revealing bilateral hilar and mediastinal lymphadenopathy. However, interferon gamma releasing assay was positive for tuberculosis. Indeed, in this unique individual, both sarcoidosis and tuberculosis existed concurrently [54].

Other infectious diseases can mimic sarcoid-like reactions. They include cutaneous leishmaniasis, leprosy, and paracoccidioidomycosis [55]. Cultures or immunohistochemical staining or both may be required to distinguish these infections from sarcoidosis or sarcoid-like reaction.

#### 3.8.3. Lymph Node Lesions: Metastatic Melanoma or Sarcoidosis or Both 

In oncologic patients with metastatic melanoma, sarcoidosis can simultaneously occur in the lymph nodes and lungs and thereby mimic metastatic neoplastic disease [22]. A 35-year-old man with metastatic melanoma presented with mediastinal lymphadenopathy. In addition, several lymph nodes in his left groin were enlarged. Ultrasound guided biopsy of a left inguinal node showed a non-caseating granulomatous "gigantoepithelioid" infiltrate. However, complete lymph node dissection of his left groin revealed not only sarcoidal granulomas, but also focal lymph node melanoma metastasis [18]. Similarly, another oncologic patient, a 52-year-old man with metastatic melanoma, developed alpha-interferon-induced sarcoidosis that was indistinguishable from melanoma metastases on positron emission tomography scan; multiple biopsies were required to confirm the presence of sarcoidal granulomas and exclude metastatic disease [36]. 

## 4. Conclusions

Sarcoidosis is a systemic inflammatory disease characterized by the development of noncaseating granulomas in one or multiple organs. Pulmonary and cutaneous manifestations are most common. However, any organ system, including the central nervous system, can be affected.

Patients with hematologic malignancies or solid tumors, including melanoma, may be at an increased risk of developing sarcoidosis. Moreover, evidence from epidemiologic studies indicates that malignancies are more common among patients who were previously diagnosed with sarcoidosis. Various antineoplastic therapies—including interferons and ipilimumab—may also induce sarcoidosis in some patients with melanoma.

The apparent association between melanoma and sarcoidosis is suggestive of a common etiologic factor. Some investigators have hypothesized that ultraviolet light plays a key role in activating an inflammatory cascade that contributes to both sarcoidosis and melanoma. However, a definitive mechanism of pathogenesis remains to be established.
